# Suicide-Related Internet Searches During the Early Stages of the COVID-19 Pandemic in the US

**DOI:** 10.1001/jamanetworkopen.2020.34261

**Published:** 2021-01-21

**Authors:** John W. Ayers, Adam Poliak, Derek C. Johnson, Eric C. Leas, Mark Dredze, Theodore Caputi, Alicia L. Nobles

**Affiliations:** 1 Division of Infectious Diseases and Global Public Health, Department of Medicine, University of California San Diego, La Jolla; 2 Department of Computer Science, Barnard College, Columbia University, New York, New York; 3 Department of Family Medicine and Public Health, Division of Health Policy, University of California San Diego, La Jolla; 4 Department of Computer Science, Johns Hopkins University, Baltimore, Maryland; 5 Department of Health Sciences, University of York, York, United Kingdom

## Abstract

This cross-sectional study assesses trends in suicide-related internet search rates in the early stages of the COVID-19 pandemic in the US.

## Introduction

Experts anticipate that the societal fallout associated with the coronavirus disease 2019 (COVID-19) pandemic will increase suicidal behavior, and strategies to address this anticipated increase have been woven into policy decision-making without contemporaneous data.^1,2^ For instance, President Trump cited increased suicides as an argument against COVID-19 control measures during the first presidential debate on September 29, 2020.

Given the time delays inherent in traditional population mental health surveillance, it is important for decision-makers to seek other contemporaneous data to evaluate potential associations.^3^ To assess the value that free and public internet search query trends can provide to rapidly identify associations, we monitored suicide-related internet search rates during the early stages of the COVID-19 pandemic in the US.

## Methods

In this cross-sectional study, we replicated the methods of a previous study on tracking suicidal ideation.^4^ Weekly Google search rates (per 10 million searches) for the term *suicide* after excluding searches mentioning *squad* (a reference to a popular film) that originating from the US between January 1, 2010, and July 5, 2020, were monitored using the Google Trends application programming interface (Alphabet Inc). We also monitored the top 20 unique queries related to suicide after unrelated terms (eg, *suicide slide*) had been excluded. This study was exempted from ethical review and certified as not qualifying as human participant research by the University of California San Diego Human Research Protections Program. This study followed the Strengthening the Reporting of Observational Studies in Epidemiology (STROBE) reporting guideline.

Changes in search rates were compared before and after the US declaration of a national emergency for the COVID-19 pandemic during the second week of March 2020. Search rates between January 1-7, 2010, and March 1-7, 2020, were used to forecast expected search rates between March 8-14, 2020, and July 5-12, 2020, using an autoregressive integrated moving average model. The ratio of observed and expected search rates with bootstrapped 95% CIs was computed weekly and cumulatively. Absolute volume of searches was estimated by multiplying the search rates by total search estimates from comScore.com (comScore Inc). Analyses were performed using R, version 3.6.1 (R Project for Statistical Computing)

## Results

**Figure 1.**
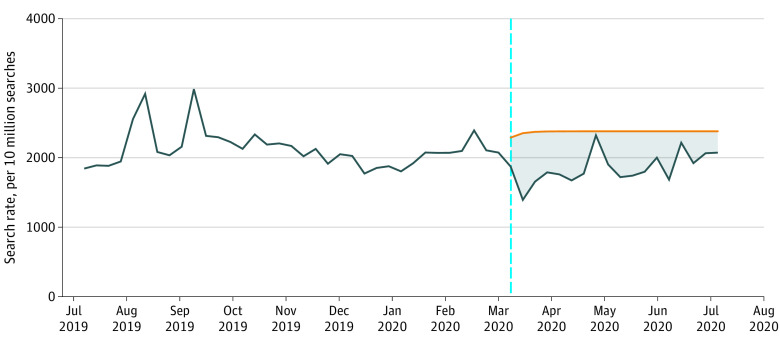
Suicide-Related Internet Searches During the COVID-19 Pandemic in the US from July 2019 through July 2020 Values are the observed rate (blue line) of all Google searches including the term *suicide* (excluding searches also mentioning *squad* representing searches for the movie *Suicide Squad*) and expected search rates (projection of historical trends; orange line) after the Trump Administration’s declaration of a national emergency in response to the COVID-19 pandemic (dashed line). The shaded area represents differences between the observed and expected search rates.

All queries containing the term *suicide* cumulatively decreased by 22% (95% CI, 18%-26%) in the 18 weeks after President Trump declared a national emergency and never eclipsed their expected search rate for any week (Figure 1). In raw terms, this was approximately 7.8 million fewer searches than expected.

**Figure 2.**
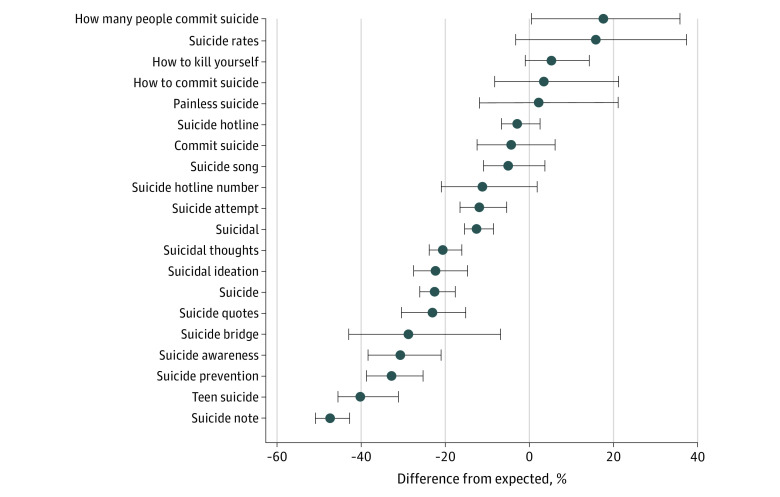
Difference Between Observed and Expected Search Rates During the COVID-19 Pandemic in the US Values are the cumulative excess for the 20 suicide-related searches with corresponding 95% CIs from March 8, 2020, through July 5, 2020.

Moreover, searches for 15 of the 20 related terms significantly decreased, including *suicide note* (–47%; 95% CI, −52% to −43%), *suicidal thoughts* (−20%; 95% CI, −24% to −16%), and *suicidal ideation* (−22%; 95% CI, −28% to −15%), translating into approximately 245 000, 155 000, and 80 000 fewer searches than expected, respectively (Figure 2). The only search term that significantly increased was potentially associated with interest in suicide facts: *how many people commit suicide* (18%; 95% CI, 1%-36%; approximately 13 000 more searches than expected).

## Discussion

Internet searches for suicide, previously found to be associated with population changes in suicidal behavior,^3,5^ decreased during the early stages of the COVID-19 pandemic in the US. Although this study cannot independently confirm that changes in search rates were caused by changes in population-level suicide rates, it showed that COVID-19 may have been inversely associated with population suicide trends between March and July 2020. Counter to expectations, our early findings are supported by the literature on catastrophic events.^6^ In some cases, catastrophes are associated with increased social support and unify communities and are thereby associated with reduced suicidal outcomes (what experts call the “pulling together” phenomenon). These include acute events, such as the 1995 Great Hanshin-Awaji Earthquake, and long-lasting events, such as the response to the September 11 attacks on the US and the subsequent recovery efforts, and now potentially the COVID-19 pandemic. For instance, at the onset of the COVID-19 outbreak, business and community leaders adopted the United Nations’ “We are all in this together” campaign, a theme that has since permeated pop culture.

Still, search rates for information on suicide may change, even increase, especially given a prolonged pandemic, making continued monitoring crucial. Moreover, researchers can extend the approach that we used (including tracking online help-seeking searches and social media shares) to empirically assess complementary proxies for other population mental health outcomes. Decision-makers could track hundreds of mental health search queries, identify the subsets that have greater demand, and target resources to meet those needs. Timely, empirical evidence from contemporaneous digital data sources can help steer limited resources to align with the needs of the public and promote data-driven debate regarding the potential societal implications of the COVID-19 pandemic.
